# Revolutionizing Dental Imaging: A Comprehensive Study on the Integration of Artificial Intelligence in Dental and Maxillofacial Radiology

**DOI:** 10.7759/cureus.50292

**Published:** 2023-12-10

**Authors:** Alekhya G iruvuri, Gouthami Miryala, Yusuf Khan, Nishaalini T Ramalingam, Bharath Sevugaperumal, Mrunmayee Soman, Aishwarrya Padmanabhan

**Affiliations:** 1 General Dentistry, Malla Reddy Dental College for Women, Hyderabad, IND; 2 General Dentistry, SVS Institute of Dental Sciences, Mahabubnagar, IND; 3 Orthodontics and Dentofacial Orthopaedics, Diamond Medical Specialists, Taif, SAU; 4 General Dentistry, Government of the Northwest Territories, Yellowknife, CAN; 5 General Dentistry, Rajah Muthiah Dental College and Hospital, Annamalai University, Chidambaram, IND; 6 Dentistry, Dr. D. Y. Patil Dental College and Hospital, Pune, IND; 7 Orthodontics, Sri Ramakrishna Dental College, Jaunpur, IND

**Keywords:** precision dentistry, maxillofacial radiology, diagnostic accuracy, deep-learning, dental imaging, convolutional neural networks, artificial intellingence in dentistry

## Abstract

Recent advancements in deep learning and artificial intelligence (AI) have profoundly impacted various fields, including diagnostic imaging. Integrating AI technologies such as deep learning and convolutional neural networks has the potential to drastically improve diagnostic methods in the field of dentistry and maxillofacial radiography. A systematic study that adhered to Preferred Reporting Items for Systematic Reviews and Meta-Analyses (PRISMA) standards was carried out to examine the efficacy and uses of AI in dentistry and maxillofacial radiography. Incorporating cohort studies, case-control studies, and randomized clinical trials, the study used an interdisciplinary methodology. A thorough search spanning peer-reviewed research papers from 2009 to 2023 was done in databases including MEDLINE/PubMed and EMBASE. The inclusion criteria were original clinical research in English that employed AI models to recognize anatomical components in oral and maxillofacial pictures, identify anomalies, and diagnose disorders. The study looked at numerous research that used cutting-edge technology to show how accurate and dependable dental imaging is. Among the tasks covered by these investigations were age estimation, periapical lesion detection, segmentation of maxillary structures, assessment of dentofacial abnormalities, and segmentation of the mandibular canal. The study revealed important developments in the precise definition of anatomical structures and the identification of diseases. The use of AI technology in dental imaging marks a revolutionary development that will usher in a time of unmatched accuracy and effectiveness. These technologies have not only improved diagnostic accuracy and enabled early disease detection but have also streamlined intricate procedures, significantly enhancing patient outcomes. The symbiotic collaboration between human expertise and machine intelligence promises a future of more sophisticated and empathetic oral healthcare.

## Introduction and background

In recent years, remarkable progress in artificial intelligence (AI) and advanced neural networks has transformed numerous fields, including speech, vision, robotics, natural language processing, and machine learning [1]. Deep learning (DL), a subset of machine learning, has become particularly crucial in diagnostic imaging. DL AI systems, also known as deep neural networks, possess the ability to learn from training data by extracting features and interpreting test data without explicit instructions [2,3]. These systems, particularly convolutional neural networks (CNNs), are used for complicated pictures produced by diagnostic imaging techniques such as cone beam computed tomography (CBCT) and magnetic resonance imaging (MRI) [4].

AI systems can currently diagnose diseases, forecast treatment outcomes, segment images, and classify them at a level that is comparable to or even better than human performance [5]. Computer-aided diagnosis is a phrase used to describe the phenomenon wherein machines correct human mistakes made during diagnosis as a result of technological improvement. To detect dental caries, periapical and periodontal diseases, root fractures, osteoporosis, cysts, and tumours in the jaws, advanced CBCT images are combined with a variety of conventional radiographs [6], including periapical, bitewing, panoramic, and lateral cephalometric images. These paradigm changes are being brought about by the incorporation of AI into diagnostic imaging.

Research consistently indicates that AI systems provide elevated accuracy, speed, and reliability in diagnostics, resulting in reduced manual effort and time. AI demonstrates proficiency in swiftly analyzing extensive medical data, recognizing complex patterns in images, and improving diagnostic precision, especially in identifying subtle changes. The consistency of AI, unaffected by fatigue, guarantees standardized outcomes across diverse cases, mitigating human errors. Despite not replacing human expertise, AI serves as a complement, promoting a collaborative and streamlined diagnostic process and enabling healthcare professionals to concentrate on intricate aspects of patient care [7,8].

The effective application of AI in clinical practice, however, confronts challenges. The requirement for large, precisely labelled datasets for training, verifying, and testing AI systems is a significant barrier [9]. Proper interpretation of these datasets, even for experienced radiologists, remains an aspirational goal. Additionally, the reliability of AI results, especially for tasks involving human judgement, can be challenging to comprehend and justify [10]. Privacy concerns arise as terabytes of patient data are shared for AI system development without adequate guarantees of patient information protection. Ethical considerations also loom large, as there are currently no specific laws governing AI development and its application. Moreover, there is a significant risk of bias in AI studies, making it difficult to quantify from the initial data selection to the final result interpretation [11].

Machine learning, a fundamental component of AI, plays a pivotal role by enabling computer models to learn and predict by recognizing patterns, akin to how radiologists refine their skills through continuous examination of medical images. AI diagnostic models have showcased their ability to identify various illnesses that may not be easily detected through routine conventional diagnostic procedures. These models excel in recognizing subtle patterns and anomalies in medical imaging; contributing to the early detection of conditions such as lung nodules, colon polyps, brain aneurysms, prostate cancer, and coronary artery calcifications; and even analyzing bone age [8]. While traditional diagnostic methods remain crucial, the nuanced capabilities of AI provide an additional level of sensitivity and precision, facilitating the detection of abnormalities that could be challenging or overlooked using conventional approaches. The incorporation of AI in diagnostics broadens the scope of early identification and diagnosis, potentially leading to enhanced patient outcomes by addressing these conditions in their early stages [10,12].

In the field of dental and maxillofacial radiology (DMFR), pre-clinical studies have shown promising results in AI diagnostic models, accurately locating root canal orifices, detecting vertical root fractures, and identifying proximal dental caries [13]. These initial findings have spurred further investigations aiming to translate pre-clinical successes into clinical applications [13]. Despite this progress, there has been no comprehensive assessment of the available evidence regarding AI's utilization in DMFR and diagnostic imaging [14].

AI's integration into healthcare, particularly through radiology, signifies a significant advancement. Machine learning algorithms' continuous learning capabilities promise more accurate and efficient diagnoses. Furthermore, AI's potential in DMFR presents exciting prospects for the future of oral healthcare [15]. By providing a thorough update on the most recent diagnostic skills of AI in DMFR and diagnostic imaging, this systematic study seeks to close existing knowledge gaps. This study aims to shed light on the transformative impact of AI on dentistry and oral healthcare, signifying a potential revolution in the identification and treatment of oral issues. The incorporation of AI into dental diagnostics holds the promise of substantial advancements in oral healthcare practices, representing a significant leap forward in medical diagnostics.

However, amidst the anticipated benefits, there is a critical need to address concerns related to the security and privacy of patient data. AI systems often rely on extensive datasets, including sensitive patient information, introducing a risk of data breaches and unauthorized access. This insecurity surrounding patient data poses a substantial challenge to the widespread adoption of AI in dentistry [16]. Inadequate data protection measures could lead to unauthorized access, misuse, or theft of patients' personal and health information. Given the vast amount of data utilized for AI algorithms, establishing robust cybersecurity measures becomes imperative. The potential compromise of patient data raises ethical concerns and undermines patient trust in the healthcare system [15,17].

Moreover, addressing the potential consequences of data loss is crucial. If patient data is not securely stored, there is a risk of accidental deletion, resulting in the loss of valuable health records. This loss could disrupt the continuity of care, impede accurate diagnoses, and compromise the overall effectiveness of treatment plans. To fully realize the benefits of AI in dentistry, strict implementation of data protection measures, encryption protocols, and access controls is imperative. Healthcare providers and technology developers must prioritize the establishment of secure and compliant systems to safeguard patient information. Effectively addressing these security concerns is instrumental in fostering trust among patients, practitioners, and stakeholders, ensuring that the integration of AI in oral healthcare aligns with ethical standards and privacy regulations [17].

This study's primary objective is to conduct a thorough examination of the existing literature on AI applications in DMFR. Technology based on AI has significantly changed a variety of industries, but it has had a particularly significant impact on the medical field because it automates processes including identifying anomalies, diagnosing illnesses, and determining prognoses [16]. AI's capacity to handle digitally encoded pictures for in-depth analysis and interpretation has been particularly advantageous for the field of radiology [17,18].

## Review

Methods

The systematic review followed the Preferred Reporting Items for Systematic Reviews and Meta-Analyses (PRISMA) standards and reported findings. The accuracy of diagnostic tests is expanded by PRISMA. Before the study began, the review technique was created in line with the Cochrane Handbook for Systematic Reviews of Interventions.

Focused Question

The primary topic of the systematic study on the usage and effectiveness of AI software in DMFR may be: "What is the effectiveness and application of AI in dental and maxillofacial radiology, as demonstrated in existing literature?" Using information from existing research and scholarly work, this inquiry aims to investigate the applicability and influence of AI on this particular subject.

Search Strategy

Our research methodology employed an interdisciplinary approach, encompassing cohort studies, case-control studies, and randomized clinical trials to explore the impact of platform switching on clinical outcomes and its correlation with bone level alterations. To gather relevant data, we meticulously reviewed original research articles, review articles, bibliographies, and citations.

Our search strategy involved comprehensive searches in databases such as MEDLINE/PubMed, Cochrane Central Register of Controlled Trials, and EMBASE, covering articles published from January 1, 2009 to September 23, 2023 without language or publication year restrictions. In August 2018, relevant papers were found by an electronic search of a number of databases, including PubMed, EMBASE, Medline through Ovid, Cochrane Central Register of Controlled Trials, and Scopus.

The search keywords were generated using an internet resource by combining Medical Subject Headings (MeSH) terminology particular to each database, with adjustments made for vocabulary and syntax consistency. They are as follows: (((("artificial intelligence” OR “machine learning” OR “deep learning”) AND (“cbct” OR “cone beam computed tomography” OR “radiography” OR “Orthopantograph” ) AND “dentistry”) AND ((y_10[Filter]) AND (English)).

The publication era was not limited in our search; however, we concentrated on clinical research written in English and avoided experimental publications using in vitro, ex vivo, or animal models. Duplicates were eliminated during title screening by managing the collected records with reference management software. The reference lists of this review were taken into account as we manually searched key DMFR publications.

Two reviewers separately checked the titles of every record, and they looked over the abstracts to find studies that needed to be read in full. Any discrepancies were resolved through discussion, and selected full texts were independently verified for inclusion by a third reviewer. This rigorous methodology ensured the comprehensive and systematic gathering of pertinent literature for our review.

**Figure 1:**
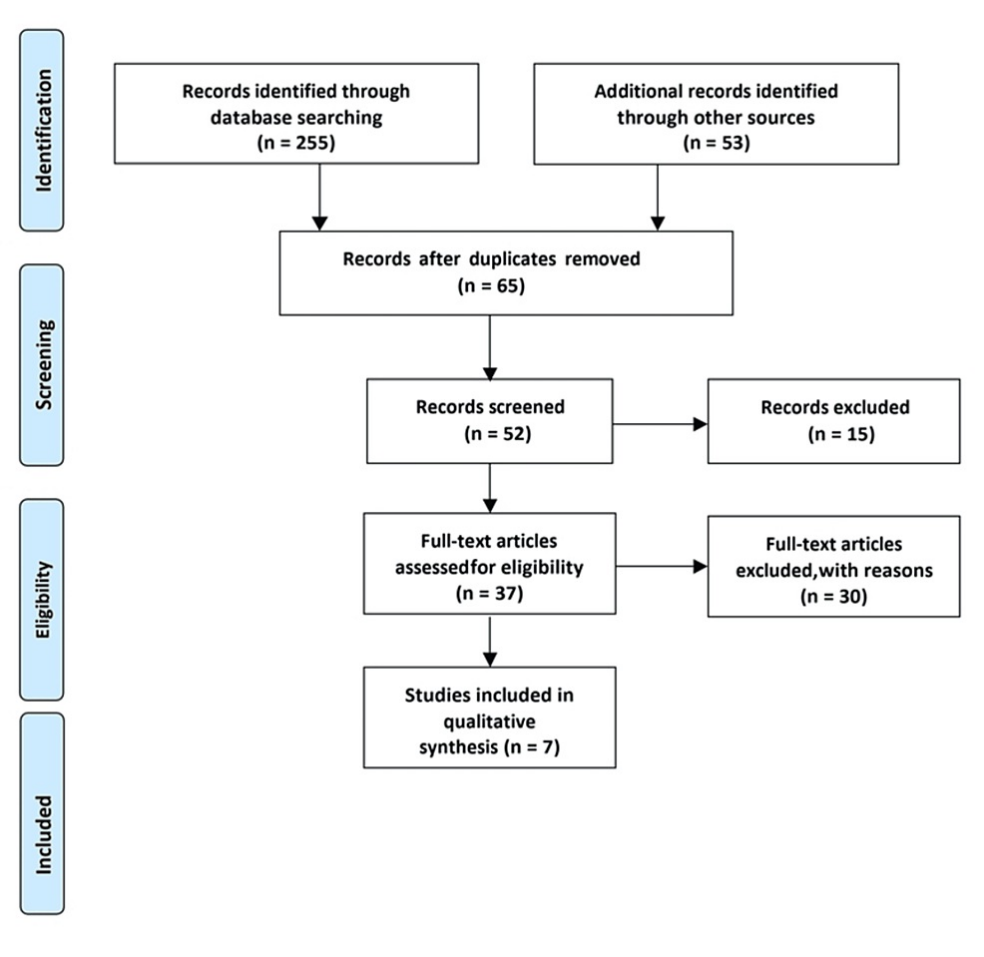
PRISMA diagram detailing the study identification and selection process. PRISMA: Preferred Reporting Items for Systematic Reviews and Meta-Analyses.

The criteria for selecting and excluding studies were established based on the PRISMA checklist. Researchers carefully examined the complete texts of the studies and evaluated them independently using predetermined inclusion criteria. Adherence to the PRISMA statement guidelines and the predefined search strategy mentioned in Figure 1 was ensured. Furthermore, a manual search was performed in the reference sections of the included studies (cross-referencing) to guarantee comprehensive coverage of the literature.

The strict inclusion requirements for the selected research included the following: (1) original publications published in English; (2) clinical studies in the field of radiology/imaging using AI models for the identification of anatomical structures in the dental and maxillofacial regions, measurement of pathological area/volume, and detection of abnormalities/pathologies; and (3) studies allowing for the evaluation of AI model performance. Studies were instead excluded if they fit into one of the following categories: (1) case reports/case series with less than 10 cases, letters to the editor, review articles, or (2) studies without full-text availability or accessibility.

This strict use of inclusion and exclusion criteria guaranteed the selection of high-quality papers, boosting the validity and applicability of our meta-analysis results in the area of AI applications in dentistry and maxillofacial radiology.

The determination of inclusion and exclusion criteria was guided by the aspects of Population, Intervention, Comparison, Outcomes, and Study design (PICOS), as shown in Table 1.

**Table 1:** The PICO framework focuses on the Population, Intervention, Comparison and Outcomes, and it is a commonly used tool for quantitative systematic review. PICO: Population, Intervention, Comparison and Outcomes; CBCT: cone beam computed tomography; AI: artificial intelligence.

Components of PICOS criteria	Explanation of the component as per our study
Population	Images captured from individuals in the oral and facial area for clinical purposes using CBCT
Intervention	Model for diagnosis using AI algorithms
Comparison	Reference standards such as a clinical or pathological examination, expert opinion, etc.
Outcome	Diagnostic capabilities of the suggested AI model, including precision, sensitivity, specificity, and mean deviation from the benchmark
Study design	Randomized clinical trials, observational study

Screening and Selection

Two authors collaborated in the search and screening process, achieving a substantial level of agreement with a κ value of 0.83. The evaluation of articles occurred in four distinct stages. In Stage 1, irrelevant citations were promptly excluded. Stage 2 involved a thorough review of titles and abstracts by one reviewer, leading to the exclusion of articles that clearly did not meet predefined criteria. For uncertain cases, a second reviewer's input was sought.

Advancing to Stage 3, articles selected in Stage 1 were independently examined by two reviewers to ensure compliance with eligibility criteria. This phase entailed excluding articles with inappropriate study designs, inadequate measurement of outcomes at both the study's outset and conclusion, and those with referencing deficiencies. Finally, in Stage 4, articles considered suitable for inclusion underwent a detailed review. Pertinent data were extracted, and the clinical methodologies of all studies were critically assessed, with a specific focus on the interventions and outcomes investigated in each study.

Data Extraction

The main author initially conducted the data extraction process, and the second author later reviewed and improved it. Data extraction was independently performed for each full-text article that met the predefined inclusion criteria. This process followed a standardized format using digital tools like Microsoft Office Excel 2013 software, as shown in Table 2. The gathered data were systematically organized into separate sections, covering information such as authorship and publication year, study design, participant demographics, age range, details of interventions, elements used for comparison, and the resulting outcomes.

**Table 2:** Data extraction sheet. CBCT: cone beam computed tomography; AI: artificial intelligence; LS: level set; DL: deep learning; PLs: periapical lesions; IoU: Intersection over Union; HD: Hausdorff distance; DSC: Dice similarity coefficient; MCD: mean curve distance; ASSD: average symmetric surface distance; RHD: robust Hausdorff distance.

Study	Population	Type of the study	Age range of patients	Parameters checked	Intervention	Comparison	Outcome
Zheng et al. [19]	180 Patients with CBCT scans were retrospectively included	Retrospective observational study	10 and 60 years old	Image segmentation using DL and LS techniques. Gender difference in pulp chamber volume. Volume difference between maxillary and mandibular molars.	Development and application of an integrated DL and LS method for the segmentation of pulp chambers in CBCT images and subsequent age estimation based on the calculated pulp volumes.	The comparison between the estimated and true human ages showed no significant difference, indicating the accuracy of the age estimation method. Despite differences in pulp chamber volume, the age estimation remained accurate.	Successful segmentation of pulp chambers in CBCT images. Comparison of gender differences in pulp chamber volume. Comparison of volume differences between maxillary and mandibular molars.
Setzer et al. [20]	Deidentified CBCT volumes from 20 patients	Observational study	19-71 years old, with a mean age of 44.4 years	Lesion detection accuracy, including sensitivity, specificity, positive predictive value, and negative predictive value, for CS and DL approaches.	Application of a DL algorithm for the segmentation and detection of PLs in CBCT images.	Demonstrated that DLS achieved excellent lesion detection accuracy with high sensitivity, specificity, positive predictive value, and negative predictive value.	These measures assessed the performance of the DL algorithm in detecting PLs in CBCT images.
Chen et al. [21]	60 patients with unilaterally impacted maxillary canines and healthy individuals	Observational study	Not mentioned	Volumetric skeletal maxilla discrepancies. The study performed linear measurements on maxillary structures to assess their dimensions.	Application of the LINKS machine learning algorithm to automatically segment maxillary structures from CBCT images.	Superimposition results highlighted variations in maxillary structures among three distinct types of impactions: buccal, mid-alveolus, and palatal.	Study tended to have smaller width, height, and depth measurements compared to control, with significant differences observed in width, height, and depth.
Shujaat et al. [22]	103 scans from patients with dentofacial deformities undergoing orthognathic surgery used CBCT	Retrospective study	Not mentioned	Evaluating metrics such as precision, recall, accuracy, Dice score, IoU, and 95% HD.	Involved the application of AI models	Precision of the AI model remained consistent across different imaging modalities	AI models consistently maintained a high level of precision across different imaging modalities.
Wang et al. [23]	30 patients who had previously undergone orthodontic treatment used CBCT	Observational cross-sectional study	11-24 years, with a mean age of 14.2 years	Segmenting dental CBCT scans into three classes: jaw, teeth, and background.	Manual segmentation of dental CBCT scans into the specified classes (jaw, teeth, and background) by experienced dentists using Mimics 21.0 software.	Multiclass segmentation involved segmenting the scans into three classes (jaw, teeth, and background), while binary segmentation grouped the scans into two classes (teeth and non-teeth).	For the multiclass segmentation approach, the DSCs for jaw ranged from 0.901 to 0.968 (mean 0.934) and for teeth ranged from 0.881 to 0.971 (mean 0.945). In the binary segmentation approach, the DSCs for jaw ranged from 0.892 to 0.966 (mean 0.933) and for teeth ranged from 0.889 to 0.973 (mean 0.948).
Jaskari et al. [24]	Analysis of mandibular canal segmentation in dental CBCT scans.	Observational, comparative analysis	Not mentioned	DSC, MCD, ASSD, and RHD.	Use of voxel-level annotations in the primary test data and coarsely annotated mandibular canals in the secondary test data.	In the primary test data, the model achieved DSC scores of 0.57 for the left canal and 0.58 for the right canal. For the MCD measure, the model obtained 0.61 mm for the left canal and 0.50 mm for the right canal.	The study demonstrated improvements in the MCD and ASSD metrics.
Leonardi et al. [25]	40 healthy patients using CBCT for evaluation	Cross-sectional study	Mean age of 23.37 years	Dice score coefficient and surface-to-surface matching technique.	Utilization of a CNN-based fully automatic method for segmenting the sinonasal cavity and pharyngeal subregions in CBCT scans.	Segmentation volumes of the three-dimensional models obtained through automatic segmentation with those obtained through manual segmentation.	Variance in segmentation volumes between the two methods was within an acceptable range and did not reach a level of significance, reinforcing the reliability of the automatic segmentation method.

Assessment of Risk of Bias

To address potential bias, we used the Risk of Bias Assessment Tool for Non-randomized Studies (ROBANS) criteria, taking into account that the studies under consideration were randomized clinical trials, as detailed in Table 3. This assessment covered the following domains: (1) random sequence generation, (2) allocation concealment, (3) selective reporting, (4) other forms of bias, (5) blinding of participants and personnel, (6) blinding of outcome assessment, and (7) incomplete outcome data.

**Table 3:** Risk of bias.

Domain	Zheng et al. [19]	Setzer et al. [20]	Chen et al. [21]	Shujaat et al. [22]	Wang et al. [23]	Jaskari et al. [24]	Leonardi et al. [25]
Random sequence generation	1	1	1	2	1	1	1
Allocation concealment	1	2	2	2	2	2	1
Selective reporting	2	1	1	1	1	1	1
Other bias	2	1	2	2	1	1	2
Blinding of participants and personnel	1	2	1	1	1	2	1
Blinding of outcome assessment	1	2	3	1	1	2	1
Incomplete outcome data	1	1	1	2	1	1	2
Total	9	10	11	11	8	10	9

Studies that provided comprehensive data across all these domains were classified as having a high level of methodological rigour, as indicated in Table 3. Those that had two to three factors present were considered to have a moderate level of quality. In contrast, studies that lacked data on most factors (either none or only one) were categorized as having lower methodological quality.

Results

In the domain of dental imaging, several research studies have delved into the integration of advanced technologies such as DL, CNN, and machine learning algorithms. These cutting-edge methods aim to enhance the precision and efficiency of dental image analysis, thereby revolutionizing diagnostics and treatment planning in the field.

One notable study by Zheng et al. involved 180 patients with cone-beam computed tomography (CBCT) scans. They developed an integrated approach utilizing DL and level set (LS) techniques to segment pulp chambers in CBCT images [19]. The primary focus of this study was on age estimation, a task accomplished through the precise calculation of pulp volumes. Remarkably, despite variations in pulp chamber volumes, the age estimation method remained consistently accurate and reliable.

In another study, Setzer et al. used DL algorithms to segment and find periapical lesions (PLs) in CBCT images [20]. Their study demonstrated the exceptional accuracy of DL systems in detecting these lesions. This achievement marked a significant advancement, potentially enhancing early detection and diagnosis of dental pathologies, thereby improving patient outcomes.

Chen et al., in their 2019 study, explored the application of the LINKS machine learning algorithm for automatic segmentation of maxillary structures in CBCT images [21]. Their investigation revealed intricate variations in maxillary structures among different types of dental impactions. These insights are invaluable for understanding the complexities of dental anomalies, aiding in more precise and tailored treatment planning through machine learning algorithms.

Shujaat et al. embarked on a retrospective study in 2021, analyzing 103 CBCT scans from patients with dentofacial deformities undergoing orthognathic surgery [22]. Their study employed various evaluation metrics, including precision, recall, accuracy, Dice score, Intersection over Union (IoU), and 95% Hausdorff distance (HD). The results highlighted the consistent performance of AI models across diverse imaging modalities. This robustness implies a versatile application of AI in the analysis of dentofacial deformities, ensuring accuracy and reliability for a wide array of patients.

Another significant contribution to the field of dental imaging came from the study by Wang et al. in 2021 [23]. They focused on segmenting dental CBCT scans into distinct classes: jaw, teeth, and background. Employing both multiclass and binary segmentation approaches, their research showcased high Dice similarity coefficients (DSCs) for both jaw and teeth segmentation. These findings underscore the efficacy of advanced segmentation techniques, enabling detailed and precise analyses of dental structures.

Additionally, Jaskari et al. conducted a comparative analysis in 2020, evaluating mandibular canal segmentation in dental CBCT scans [24]. They used measures including the Robust Hausdorff distance (RHD), mean curve distance (MCD), average symmetric surface distance (ASSD), and DSC in their research. The study showed considerable advancements in metrics for MCD and ASSD, indicating improved segmentation accuracy and dependability. These developments ensure careful identification of anatomical features and represent a big step forward in dental imaging.

The utilization of a CNN-based completely automated approach for segmenting the sinonasal cavity and pharyngeal subregions in CBCT scans was also investigated in a cross-sectional study by Leonardi et al. in 2021 [25]. The Dice score coefficient and surface-to-surface matching approach were used in their research to demonstrate how consistently the system performed with manual segmentation. This work emphasized automated segmentation's dependability and showed its potential for swiftly and precisely segmenting complex anatomical areas.

Collectively, these studies show how cutting-edge technology has completely changed dental imaging. These techniques forecast a future in which dental diagnoses and treatment planning will be not only more effective but also amazingly exact and trustworthy. They range from accurate age prediction and early lesion identification to precise segmentation of dental structures and complicated anatomical areas.

Discussion

Dental imaging diagnostics have seen a radical transformation thanks to the integration of cutting-edge technologies such as CNNs, DL, and machine learning. This integration offers far increased precision over conventional approaches, which is a huge step forward. Deciphering the intricacies present in dental photos is made possible by DL, which is renowned for its capacity to recognize and comprehend complicated patterns in data. Because of its depth, it may identify minute characteristics that conventional diagnostic techniques frequently overlook, allowing for a more complete assessment of dental diseases as well as early identification and treatment.

CNNs, which are experts in image processing, take these developments a step further by precisely analyzing certain patterns and structures found in dental pictures through the extraction of fine-grained features from images. This greatly improves diagnostic precision, particularly when identifying anomalies or minute indications of tooth problems. Workflows are further streamlined by using machine learning algorithms, which automate monotonous operations, shorten analysis times, and adjust to changing datasets. This improves overall efficiency as well as the accuracy of the diagnosis. Dental practitioners' workflows are streamlined by real-time decision assistance, automated structure segmentation, and rapid analysis, which may lead to better patient outcomes through prompt treatments.

Zheng et al.'s work from 2021 demonstrated the accuracy and dependability that may be attained using advanced computational techniques. In order to determine age using pulp chamber contents, the researchers used likelihood-based and DL methods [19]. This method has significant implications for forensic dentistry and age-related dental treatments, in addition to offering insightful information on age-related changes in teeth. Precise age determination by computational methods can provide vital data for forensic examinations and help with customized dental procedures depending on age-related factors.

DL techniques for the identification of PLs in CBCT images were first presented by Setzer et al. in 2020 [20]. Research highlights the clinical value of these algorithms in facilitating accurate and timely diagnosis of dental diseases, emphasizing their high sensitivity and specificity. By detecting dental abnormalities at an early stage, this technological development not only makes fast therapies possible but also improves patient outcomes.

Chen et al. autonomously segmented maxillary components from CBCT pictures in 2019 using the LINKS machine learning algorithm [21]. With the use of this cutting-edge method, complex differences in maxillary structures between various dental impactions were revealed. These discoveries are essential for customized maxillofacial and orthodontic surgery planning, which improves intervention accuracy. The use of machine learning in segmentation leads to a more thorough comprehension of maxillary anatomy, facilitating customized treatment plans for individuals with various dental disorders.

The study conducted in 2021 under the direction of Shujaat et al. demonstrated the adaptability of AI models in the analysis of dentofacial abnormalities using a range of imaging modalities [22]. These models consistently perform well in sophisticated clinical settings, which improves patient satisfaction and treatment efficacy, especially in circumstances that call for elaborate treatment strategies. By utilizing AI, medical professionals can get a more sophisticated comprehension of dentofacial abnormalities, which can result in more efficient treatment plans and better results for patients undergoing extensive dental procedures.

High DSC for the segmentation of the jaw and teeth in Wang et al.'s 2021 study [23] showed the efficacy of sophisticated segmentation approaches in dental imaging. Precise segmentation is essential to prosthodontics, maxillofacial surgery, and orthodontic treatment planning, which raises the standard of treatment planning. By optimizing treatment results, the accuracy attained through sophisticated segmentation algorithms guarantees that therapies are tailored to the unique anatomical characteristics of each patient.

Mandibular canal segmentation is a crucial component of dental imaging, and Jaskari et al. focused on it in 2020 [24]. Increased accuracy is suggested by improvements in MCD and ASSD metrics. Accuracy is crucial in oral surgery and implantology to avoid problems during treatments. For dental implants to be placed securely and precisely, the mandibular canal must be segmented accurately. This reduces the possibility of difficulties and improves the overall outcome of oral surgical treatments.

A completely automated method based on CNNs was presented by Leonardi et al. in 2021 for the segmentation of pharyngeal and sinonasal cavity subregions in CBCT images [25]. The dependability shown is in line with manual segmentation techniques, which emphasizes how important correct segmentation is in a variety of dentistry and medical disciplines. Planning and carrying out therapies in intricate anatomical areas require accuracy and efficiency, which the automated segmentation approach ensures in clinical settings.

One of the most significant limitations is the dependence on both the amount and quality of data. For these advanced technologies to be trained effectively, huge and diverse datasets are essential. These models may show decreased accuracy or even reinforce biases found in the data in situations where the data may be incomplete, skewed, or not representative of the whole population.

Furthermore, there is still work to be done on making DL models interpretable. Trusting and comprehending the outputs of these systems might be difficult for physicians due to the complexity of understanding how and why these systems arrive at specific diagnoses or choices. In some therapeutic situations where transparency is crucial, the adoption of these technologies may be impeded by their lack of interpretability.

It is critical to address patient security and safety issues. Sophisticated technology must be integrated, which means that strong data protection protocols are required. It is crucial to protect patient data against misuse, illegal access, and data breaches. Strict access restrictions, encryption, and compliance with privacy laws are essential for guaranteeing the security and confidentiality of patient data.

Regarding the advantages, research carried out by different writers highlights how these technologies are revolutionizing dental diagnosis and treatment. Age-related dental treatment customization and forensic investigations both greatly benefit from the accuracy and dependability demonstrated by age assessment using computational approaches. By facilitating prompt therapies, the early identification of PLs via DL algorithms greatly enhances patient outcomes. Furthermore, the automatic segmentation methods shown in many publications have numerous advantages. They help to improve treatment results in the fields of dental implantology, maxillofacial surgery, and orthodontics by facilitating individualized treatment planning and improving the precision of procedures. 

The AI models' capacity to improve treatment efficacy and patient happiness is further shown by their consistency and dependability in assessing intricate dental circumstances. Thus, even though these cutting-edge technologies have the potential to revolutionize dental imaging, their successful integration into clinical practice depends on recognizing their limitations, protecting patient data, and highlighting their substantial benefits in precision diagnosis and customized treatments.

## Conclusions

In summary, the incorporation of DL, CNNs, and machine learning algorithms into dental imaging stands as a remarkable leap in the field, heralding an era of unmatched precision and efficiency. These state-of-the-art technologies have transformed how dental professionals interpret images, enabling them to make highly precise diagnoses, pinpoint subtle irregularities, and design customized treatments with unprecedented accuracy. By leveraging AI, dental imaging has transcended its conventional limitations, empowering clinicians to enhance decision-making, simplify intricate procedures, and ultimately improve patient outcomes significantly. This transformative change not only raises the bar for healthcare standards but also ushers in a realm of innovative research and development, promising a future where dental diagnostics and therapies are not only more effective but also increasingly tailored and patient-centered.

The impact of DL and its allied technologies in dental imaging goes well beyond technical advancement; it signifies a fundamental shift in how oral health is approached and managed. As these advanced algorithms continue to progress, dental professionals can anticipate even more sophisticated diagnostic abilities, facilitating early disease detection, precise treatment strategizing, and enhanced patient experiences. The integration of AI into dental imaging not only enhances the skills of practitioners but also exemplifies the harmony between human expertise and machine intelligence. This synergy, where knowledge and computational capabilities converge, becomes a transformative catalyst for shaping the landscape of dental healthcare. Embracing these technologies not only ensures superior clinical outcomes but also sets the stage for a more nuanced and compassionate approach to oral health, marking a significant milestone in the evolution of dental practice.
